# Field Evaluation of Synthetic Components of the Sex Pheromone of the Tea Pest *Helopeltis cinchonae* Mann (Hemiptera: Miridae)

**DOI:** 10.3390/insects16030307

**Published:** 2025-03-16

**Authors:** Fida Hussain Magsi, David R. Hall, Zongxiu Luo, Xiangfei Meng, Chunli Xiu, Zhaoqun Li, Lei Bian, Nanxia Fu, Jianlong Li, Zongmao Chen, Xiaoming Cai

**Affiliations:** 1 Key Laboratory of Tea Biology and Resource Utilization, Ministry of Agriculture, Tea Research Institute, Chinese Academy of Agricultural Sciences, Meiling South Road 9, Xihu District, Hangzhou 310008, China; fidajanmagsi@yahoo.com (F.H.M.); luozongxiu@tricaas.com (Z.L.); 15137389353@163.com (X.M.); xiuchunli@tricaas.com (C.X.); zqli@tricaas.com (Z.L.); bianlei@tricaas.com (L.B.); funanxia@tricaas.com (N.F.); 2 Natural Resources Institute, University of Greenwich, Chatham Maritime, Kent ME4 4TB, UK; 3 Tea Research Institute, Guangdong Academy of Agricultural Sciences, No.6 Dafeng Road, Wushan, Tianhe District, Guangzhou 510640, China; skylong.41@163.com

**Keywords:** pheromone blend, trap design, lure longevity, field trapping, monitoring

## Abstract

*Helopeltis cinchonae* is an emerging pest of tea and causes severe damage to tea plantations in China. The female of *H. cinchonae* has been reported to produce a sex pheromone consisting of two components, hexyl (3*R*)-3-acetoxybutyrate and (5*R*)-1-acetoxy-5-butyroxyhexane, and lures containing the synthetic compounds have been shown to attract male *H. cinchonae* to traps in the field. This is the first time that components of the sex pheromone have been identified for a species of *Helopeltis* bug, but their field application has not been evaluated in detail. The present study shows that a blend of both compounds loaded into a polyethylene vial at 0.2 and 2 mg, respectively, caught significantly more male *H. cinchonae* bugs than the individual compounds and all the other tested blends. Sticky wing traps baited with the binary blend of compounds at the optimized ratio and dosage caught more bugs than bucket funnel or delta traps, and traps hung at a height of 10 cm above the tea shoots caught more bugs than those at other heights. The optimized traps and lures were used for monitoring the pest and two distinct population peaks of *H. cinchonae* were observed, the first one during mid-May to early June, and the other one in mid-September. Overall, the results of this study contribute to an environmentally-friendly approach to monitoring and managing *H. cinchonae* in the field.

## Simple Summary

*Helopeltis cinchonae* is an economically important pest of tea plantations in Asia and has recently been reported in various provinces of China, expanding its population range. The bugs feed on growing shoots, sucking the sap and leaving the shoots susceptible to infection by fungi. No known control or monitoring tools have been developed for *H. cinchonae* except for the excessive application of pesticides. The components of the sex pheromone produced by female *H. cinchonae* have been identified and synthesized in previous work and have been shown to attract male bugs to traps. This is the first time that the components of the sex pheromone have been identified for a species of *Helopeltis* bug. Here, we present the results of field trapping tests to optimize the blend of pheromone components, compare different types of traps, and determine the best position of the trap in order to maximize the catch of male bugs. Traps baited with the optimized lure were used to monitor populations of the bug and showed two distinct generations during the year. These results contribute to an environmentally-friendly approach to monitoring and managing *H. cinchonae* in the field.

## 1. Introduction

*Helopeltis cinchonae* (Mann, 1907) (Hemiptera: Miridae) belongs to the Bryocorinae subfamily of mirids, distributed across Asia, Australia, Africa, and other geographical regions [1]. The major host plants of *H. cinchonae* are tea [2], cocoa [3], and cashew [4], but it has a broad range of other host crops [5,6]. Tea is a commercially important crop in China [7,8], and *H. cinchonae* causes severe damage during the whole growing season by feeding on fresh, pickable shoots [9]. Other *Helopeltis* spp. similarly damage the young shoots or fruits of various host crops [10]. The feeding damage enhances tissue infection by opportunistic fungi, and the affected leaves become deformed, curl up, and eventually die, directly affecting the next flush of tea shoots and the tea yield [11]. Consecutive outbreaks of *H. cinchonae* have occurred in Chinese tea plantations during the past few years, and the application of conventional pesticides to manage the pest is the only known control method. Excessive application of pesticides has caused resistance [12] and chemical residue problems, which are directly hazardous to livelihoods and human health [13]. Although treatment with pesticides results in the suppression of nymphs and adult populations of *H. cinchonae*, there is less impact of those pesticides on eggs [14], and thus the newly emerged nymphs start the damage and successive life cycles of the pest grow during the whole season of tea plantations. Therefore, new and environmentally-sustainable approaches to managing the pest are needed.

At present, semiochemical approaches are not being used widely to manage insect pests of tea [15]. However, these are becoming increasingly popular [15,16] and are likely to be adopted by many tea growers [17] because of their zero adverse effects on the environment as well as on the tea consumers [18,19].

The sex pheromones of more than 20 mirid species have been chemically identified and synthesized [20], and they are being used to monitor [21], disrupt [22], or manage the mirid species [23]. The sex pheromone of *H. cinchonae* was identified recently in our previous study [2] as a blend of two components, (5*R*)-1-acetoxy-5-butyroxyhexane and hexyl (3*R*)-3-acetoxybutyrate. The latter compound was identified as a potential sex pheromone component of *Macrolophus caliginosus* [24], but the former is a novel compound, identified for the first time as an insect pheromone component. In our preliminary field experiments, both compounds, as a blend or alone, attracted male *H. cinchonae*. However, the optimal ratio of sex pheromone components, trap type, trap height, and lure longevity have not been reported. This study was designed to investigate these parameters in order to develop an effective lure to monitor and manage *H. cinchonae* in tea plantations.

## 2. Materials and Methods

### 2.1. Location of Field Experiments

All the field experiments were conducted in Jiexi Guangdong, China (23.38° N, 115.5° E), during the years 2023–2024. Before any experiment, a sticky wing trap (42 cm in length × 26.5 cm in width × 16 cm in height; Pherobio Technology Co. Ltd., Beijing, China) with a white sticky board (42.7 cm in length × 28 cm in width), baited with a binary blend of *H. cinchonae* sex pheromone components, was set in the field and observed at intervals of 10 d to determine the availability of insects in the field. During the experimental period, no pesticides were applied to ensure a resident population of pests.

### 2.2. Lure Preparation

The sex pheromone components, hexyl (3*R*)-3-acetoxybutyrate and (5*R*)-1-acetoxy-5-butyroxyhexane, were synthesized as in our previous study [2]. The purity of the compounds was greater than 97%, as confirmed by gas chromatography–mass spectrometry analysis. Blends of the synthetic compounds were added to paraffin oil (400 μL; m.p. −24 °C; CAS# 8012-95-1; Shanghai Aladdin Biochemical Technology Co., Ltd., Shanghai, China) and loaded into polyethylene vials (56 × 13 × 0.25 mm thickness; Shun Xing Plastic Products Ltd., Guangzhou, Guangdong, China). The vials were heat-sealed, and the pheromone compounds diffused through the polyethylene wall. After loading, lures were kept at −20 °C until used for field tests.

### 2.3. Measurement of Release Rates

Polyethylene vials loaded with hexyl (3*R*)-3-acetoxybutyrate and (5*R*)-1-acetoxy-5-butyroxyhexane (0.2 mg and 2 mg, respectively; *N* = 3) were maintained under laboratory conditions in a wind tunnel at 26 ± 1 °C, with a wind speed of 2.3 m/sec. At 4 d intervals, the volatiles from each dispenser were collected by putting it in a glass chamber (24 cm in length × 6 cm in diameter) and drawing charcoal-filtered air (200 mL min^−1^) through the chamber for 3 h. The first volatile collection was performed two days after lure preparation. The released volatiles were trapped in a glass column (4.5 cm long × 0.8 cm diameter) containing Porapak Q (50 mg; 50–80 mesh; SigmaAldrich, Shanghai, China), then eluted with dichloromethane (200 µL) and analyzed by gas chromatography–mass spectrometry. A DB-23 column was used for these analyses, and the conditions were the same as in our previous study [2].

### 2.4. Field Experiments

#### 2.4.1. Comparison of Pheromone Blends

The first field experiment was conducted to determine the optimal ratio of *H. cinchonae* sex pheromone components during 10–17 September 2023. Sticky wing traps were baited with one of eight treatments including the control (Table 1). Traps were positioned approximately 15 cm above the top of the crop, and each treatment was replicated three times. Trap captures were recorded every two days and the white sticky boards were changed on each observation. The lures were not renewed, and polyethylene vials loaded with only 400 μL of paraffin oil were used as control.

#### 2.4.2. Comparison of Trap Types

Three different types of traps were evaluated during 9–15 October 2023 for their trapping efficiency: (1) a sticky wing trap, (2) a bucket funnel trap (16 cm in diameter × 20 cm in height, Pherobio Technology Co. Ltd., Beijing, China), and (3) a delta trap (25 cm in length × 18 cm in width × 16 cm in height; Enjoy Technology Co, Ltd., Zhangzhou, China), with a white sticky board (24 cm in length × 16 cm in width). A polyethylene vial loaded with the blend of sex pheromone compounds, hexyl (3*R*)-3-acetoxybutyrate and (5*R*)-1-acetoxy-5-butyroxyhexane (0.2 and 2 mg, respectively), was placed in the center of the white sticky boards in the sticky wing trap and in the delta trap. For the bucket funnel trap the vial was placed in a lure holder under the lid. All three traps were installed 15 cm above the tea shoots. Each trap type was replicated five times.

#### 2.4.3. Evaluation of Optimum Trap Height

The sticky wing trap was used to evaluate the optimal trap height during 24–30 May 2024. The traps were set at three different heights, 10, 25, and 50 cm above the tea shoots. Lures consisted of polyethylene vials loaded with the blend of sex pheromone components, hexyl (3*R*)-3-acetoxybutyrate and (5*R*)-1-acetoxy-5-butyroxyhexane (0.2 and 2 mg, respectively). Each treatment was replicated four times. Catches were recorded every two days, and white sticky boards were changed after each observation.

#### 2.4.4. Population Monitoring

Three sticky wing traps baited with polyethylene vials loaded with hexyl (3*R*)-3-acetoxybutyrate and (5*R*)-1-acetoxy-5-butyroxyhexane (0.2, and 2 mg, respectively) were set in a tea field (August 2023–July 2024) 15 cm above the tea shoots and were spaced 15 m apart. Catches of *H. cinchonae* were counted every 5 d and the sticky boards were changed after each observation. Lures were renewed after 50 days. The experiment was monitored from August 2023 to July 2024, and results are presented as mean catches over 15 d periods.

### 2.5. Data Analysis

Trap catches of *H. cinchonae* were transformed by log_10_ (trap catch + 1) to ensure the homogeneity of variance and a normal distribution and then analyzed by one-way ANOVA followed by a *post hoc* Tukey’s-*b* test (*p* < 0.05). All the statistical analyses were performed using SPSS statistics 21.0 (IBM, Armon, NY, USA).

## 3. Results

### 3.1. Release Rate Measurements

**Figure 1:**
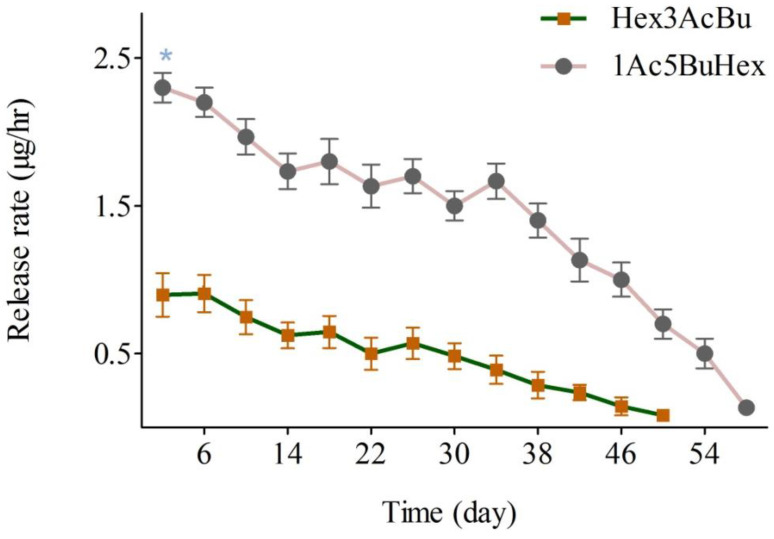
Release rates of hexyl (3*R*)-3-acetoxybutyrate (Hex3AcBu) and (5*R*)-1-acetoxy-5-butyroxyhexane (1Ac5BuHex) from polyethylene vials loaded with 0.2 mg and 2 mg, respectively, of each compound and maintained in a laboratory wind tunnel at 26 ± 1 °C and a wind speed of 2.3 m/s (mean ± SE; *N* = 3; * indicates release rate significantly higher, *p* < 0.05).

The measurement of release rates from a polyethylene vial containing the two pheromone components hexyl (3*R*)-3-acetoxybutyrate and (5*R*)-1-acetoxy-5-butyroxyhexane (0.2 mg and 2 mg, respectively) showed initial release rates of 0.90 μg/h and 2.30 μg/h, respectively (Figure 1). The release rates of both compounds gradually decreased. For hexyl (3*R*)-3-acetoxybutyrate, the lowest release recorded was 0.08 μg/h on day 50, and no traces were detected on day 56. The major component, (5*R*)-1-acetoxy-5-butyroxyhexane, was still detectable on day 58 at 0.08 μg/h (Figure 1).

### 3.2. Comparison of Pheromone Blends

**Figure 2:**
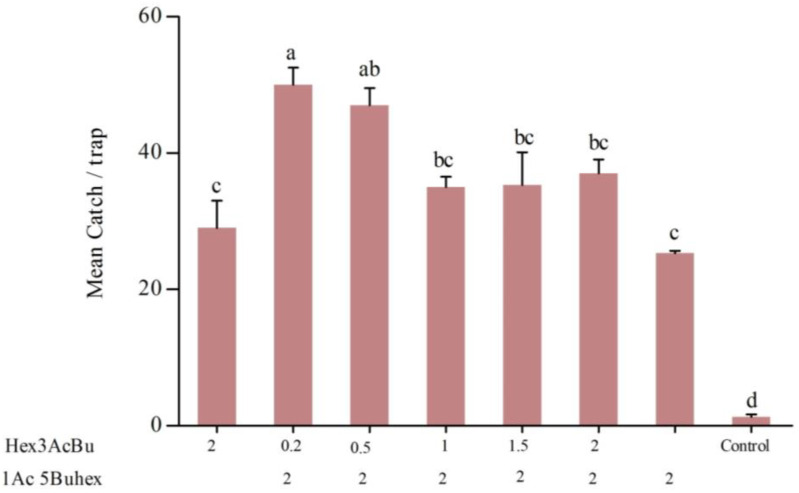
Mean catches (±SE; *N* = 3) of male *Helopeltis cinchonae* in traps baited with single or binary blends of hexyl (3*R*)-3-acetoxybutyrate (Hex3AcBu) and (5*R*)-1-acetoxy-5-butyroxyhexane (1Ac5Buhex) during 10–17 September 2023. Bars capped with lowercase letters are significantly different according to the *post hoc* Tukey’s-*b* test (*p* < 0.05).

In the first field experiment, 776 male *H. cinchonae* were caught in traps containing different ratios of the pheromone components, i.e., hexyl (3*R*)-3-acetoxybutyrate and (5*R*)-1-acetoxy-5-butyroxyhexane or the single compounds (Figure 2). Highest catches were recorded in traps baited with blends containing both compounds at 0.2 mg and 2.0 mg, followed by 0.5 mg and 2.0 mg, respectively (*F* = 30.896; df = 7,16; *p* < 0.001). Traps baited with lures containing the single compounds caught lower numbers of males, but significantly more than unbaited traps, with no significant differences in trap catches (Figure 2).

### 3.3. Comparison of Trap Types

**Figure 3:**
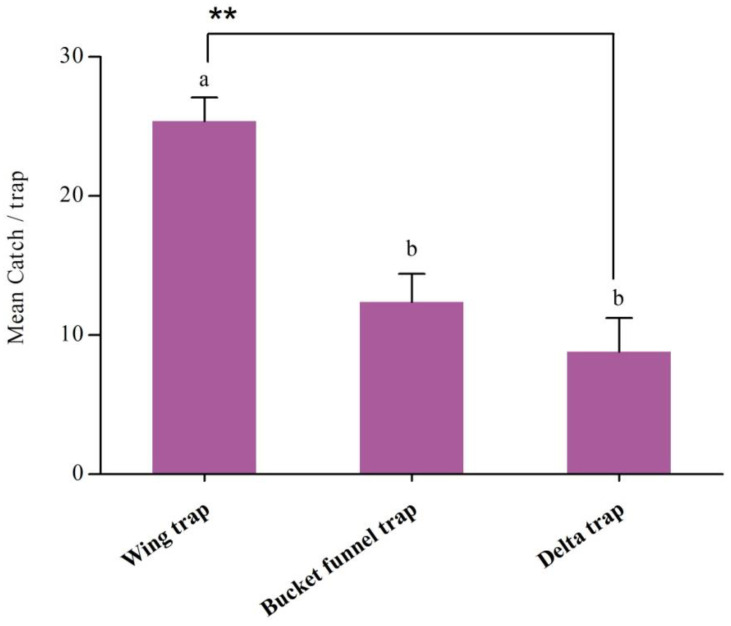
Mean catches (±SE; *N* = 5) of male *Helopeltis cinchonae* in three different trap types, baited with a binary blend of hexyl (3*R*)-3-acetoxybutyrate and (5*R*)-1-acetoxy-5-butyroxyhexane (0.2 and 2 mg, respectively) during 9–15 October 2023. Bars capped with lowercase letters are significantly different according to the *post hoc* Tukey’s-*b* test (*p* < 0.05); ** indicates catches were significantly different at *p* < 0.01.

In a comparison of three trap types, the wing trap captured significantly more male *H. cinchonae* than delta or bucket funnel traps (*F* = 17.788; df = 2,12; *p* < 0.001), and there was no difference in catches between the latter two types of traps (Figure 3).

### 3.4. Evaluation of Optimum Trap Height

**Figure 4:**
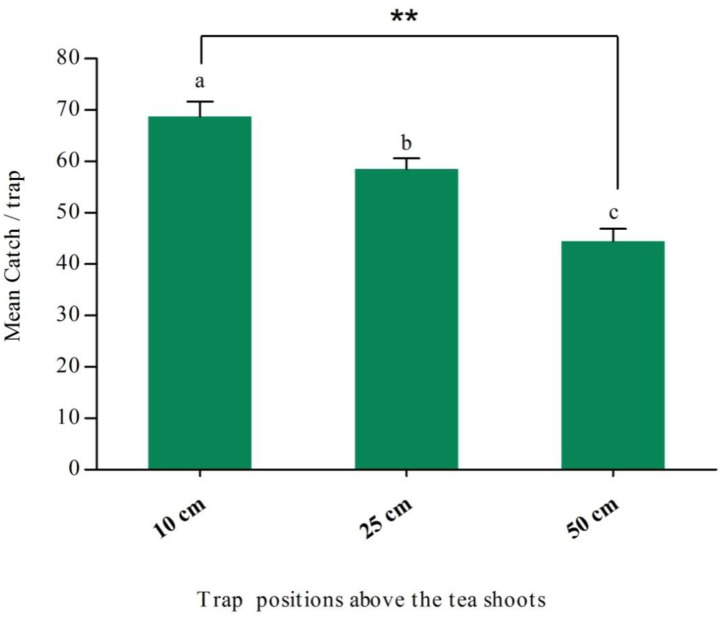
Mean catches (±SE; *N* = 5) of male *Helopeltis cinchonae* in wing traps hung at three different heights (10, 25 and 50 cm) above the crop. Traps were baited with a binary blend of hexyl (3*R*)-3-acetoxybutyrate and (5*R*)-1-acetoxy-5-butyroxyhexane (0.2 and 2 mg, respectively) during 24–30 May 2024. Bars capped with lowercase letters are significantly different according to the *post hoc* Tukey’s-*b* test (*p* < 0.05); ** indicates catches were significantly different at *p* < 0.01.

When catches of male *H. cinchonae* in wing traps positioned at three different heights, i.e., 10, 25, and 50 cm above the tea shoots, were compared, catches were significantly reduced as traps were raised higher above the crop. The highest catches were recorded in traps hung 10 cm above the tea shoots (*F* = 24.059; df = 2,9; *p* < 0.001) (Figure 4).

### 3.5. Population Monitoring

**Figure 5:**
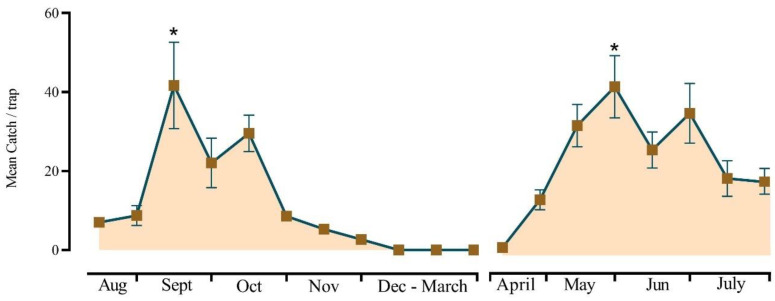
Variation in mean catches (2 August 2023–30 July 2024) of male *Helopeltis cinchonae* (±SE; *N* = 3) in sticky wing traps baited with polyethylene vials impregnated with hexyl (3*R*)-3-acetoxybutyrate and (5*R*)-1-acetoxy-5-butyroxyhexane (0.2 mg and 2 mg, respectively); * indicates catches were significantly higher on those occasions (*p* < 0.05).

Monitoring traps baited with the optimum pheromone blend showed two distinct population peaks in the tea plantations when trap catches were significantly higher than in all other months (*F* = 21.997; df = 15,32; *p* < 0.001) (Figure 5). The first peak was observed from the middle of May to early June, and a second peak occurred in mid-September with a trough in early August. Catches of *H. cinchonae* declined after this to zero at the end of November.

## 4. Discussion

Two components of the female-produced sex pheromone of *H. cinchonae* were identified previously as hexyl (3*R*)-3-acetoxybutyrate and (5*R*)-1-acetoxy-5-butyroxyhexane [2]. This was the first time components of the sex pheromone of a species of *Helopeltis* had been identified, but the field application of the pheromone was not studied in detail. Numerous factors influence trap captures and are considered essential for enhancing trapping efficiency [19,25,26]. In this study, we investigate the optimal blend of identified pheromone components, type of trap, trap height, and release rate of the pheromone and demonstrate the use of the most optimized system for monitoring the pest.

In our preliminary studies, we tested blends of the two components of the pheromone of *H. cinchonae* in two field experiments [2]. In the first of these, the lure loaded with 1.5 mg of each compound was highly attractive to male *H. cinchonae*, but, in the second field experiment, the lure loaded with 0.2 mg hexyl (3*R*)-3-acetoxybutyrate and 2 mg (5*R*)-1-acetoxy-5-butyroxyhexane attracted significantly more *H. cinchonae* than all the other blends tested. Furthermore, the 1:1 ratio of the compounds was different from that found in female body extracts, which was 1:10, respectively. In the current study, we tested a wider range of blends and confirmed that the most attractive blend was 1:10, respectively, in agreement with our second preliminary experiment and the ratio found in the female bugs. This is similar to results obtained from another mirid species, *Sahlbergella singularis* Haglund (Hemiptera: Miridae) [27], where blends of two female-produced compounds, hexyl (3*R*)-3-[(*E*)-2-butenoyloxy]-butyrate and hexyl (3*R*)-3-hydroxybutyrate, have been shown to attract more male *S. singularis* than the individual compounds [27].

The type of trap plays a key role in trapping efficiency [28]. In our trap-type experiment, the results show that the sticky wing trap type caught 60% more male *H. cinchonae* than delta or bucket funnel traps. This may be because the wing-type trap is bigger and open on all four sides, allowing the insect to enter from any side [15]. Furthermore, we noticed that male *H. cinchonae* were not landing directly near the lure but landing on the top of the trap or near it, walking on the sides of the white sticky boards and then flying away. Other mirid bugs release alarm pheromones when disturbed [29,30]. The male *H. cinchonae* which were already trapped on the white sticky boards were still alive and possibly releasing alarm pheromones which might have repelled the male *H. cinchonae* walking on the side of the white sticky boards. Although the sticky wing traps caught most male *H. cinchonae* bugs, many of the tea plantations in China are planted at high altitudes in hilly areas, and changing the white sticky boards after every few days is not feasible. The bucket funnel traps, with higher capacity, would be more appropriate for use in these regions, even though they catch fewer bugs.

Trap catch can also be increased by placing the traps at an optimal height [28]. In preliminary experiments, we noticed that the same lure and trap yielded variable trap catches of *H. cinchonae* when hung at different heights above the crop. Furthermore, adult *H. cinchonae* were observed flying in tea gardens during their peak in population size, and they flew from one tea bush to another without going much higher or moving great distances. Thus, our experiments showed that catches in pheromone traps were greatest when the traps were positioned just above the crop and were lower when hung higher. This is similar to the situation with another mirid, *S. singularis*, where catches were highest in traps in the cocoa canopy and reduced in traps positioned higher or lower [23,31]. Similar results have been found with pheromone traps for some other pest insects [25], but not all as this depends on the flying behavior of the target pest [25].

The lure with a blend of the two sex pheromone components of *H. cinchonae*, loaded into polyethylene vials using dichloromethane, lasted for 32 days under constant laboratory conditions [2]. In the present study, we used paraffin oil instead of dichloromethane and the lure lasted for 58 days. Sunflower oil was used to control the release of pheromone for *Lygus* spp. from pipette tip dispensers [32] and as a solvent in polyethylene vial lures for the mirid bug *Apolygus lucorum* Meyer-Dür (Hemiptera: Miridae) to increase lure longevity and release the compounds uniformly [20].

For other mirid bugs, it has been reported that release rates of pheromones from lures should be comparable to those from the female bugs [32,33], as the same pheromone components may be used as alarm pheromones or defensive compounds at high release rates [29,30,34]. Although we did not measure the release rates of pheromone components from *H. cinchonae* females, the lures tested here were highly attractive to males under field conditions over the lifetime of the lure, indicating that their release rate must be of the right order of magnitude. The measurements of release rates under defined laboratory conditions provide a standard against which to assess other lures and pheromone loadings in any future experiments

The fluctuation in capture of *H. cinchonae* numbers was relatable to its population dynamics. *Helopeltis* spp. were reported to start emergence in tea plantations during mid-March until the end of June [11,35], and remained until October [11]. A similar pattern was reflected in catches of *H. cinchonae* in pheromone traps. Heavy rains and other biotic and abiotic factors can influence the population dynamics of insect pests [35,36], and information on population dynamics is vital to inform effective pest management practices.

## 5. Conclusions

The present study aims to evaluate the application of components of the sex pheromone produced by female *H. cinchonae* in tea plantations. The blend of *H. cinchonae* sex pheromone components, hexyl (3*R*)-3-acetoxybutyrate and (5*R*)-1-acetoxy-5-butyroxyhexane, at a dosage of 0.2 mg and 2 mg, respectively, in a wing-type trap hung at a height of 10 cm above the tea shoots was found to be the most effective to catch male *H. cinchonae* and has potential to monitor and manage the pest in tea plantations. Mass trapping during two peaks in the population size of *H. cinchonae* in the tea plantations could result in an effective control tool, although the density of traps still needs to be determined.

## Figures and Tables

**Table 1:** Loadings of synthetic compounds evaluated in the field to determine the optimal ratio.

	Lure Loading (mg)	
Treatment	Hexyl (3R)-3-acetoxybutyrate	(5R)-1-Acetoxy-5-butyroxyhexane
T1	2	0
T2	0.2	2
T3	0.5	2
T4	1	2
T5	1.5	2
T6	2	2
T7	0	2
T8	Unbaited	

## Data Availability

Pheromone release rate data and trap catch data are available from the authors upon request.
